# The Value of Weighing Patients

**Published:** 1911-12-30

**Authors:** 


### POINTS OF INTEREST IN TUBERCULAR TREATMENT.

### The Value of Weighing Patients.

The difficulty of determining from variations in the abnormal physical signs alone whether in a given case of pleuritic effusion the fluid in the chest has spontaneously cleared up, or of deciding in a case that has been tapped whether the fluid is reaccumulating or not, is familiar to all. The abnormal physical signs to which fluid in the chest gives rise depend less upon the actual presence of fluid than upon the airlessness of the lung resulting from the compression of the pulmonary tissues produced by that fluid. If the walls of the thorax could be bulged outwards by pleuritic fluid in such a way that the underlying lung never had the air pressed out from its alveoli the effusion would not produce dulness but at the most an impairment of note, comparable with the impairment that is found in patients who have very thick chest walls as the result of either great fatness or of extreme muscular development. The reason why one of the most prominent physical signs of a pleuritic effusion is dulness to percussion over the corresponding part of the chest is, not that fluid is present, but that air is absent from the lung. The same applies to a considerable extent to the absence of vesicular murmur and to the absence of voice sounds.

Nearly all abnormal physical signs produced by effusion are negative—absence of movements, absence of resonance, absence of voice sounds, and these are all due to absence of air in the corresponding lung; the only positive signs of effusion in the thorax are filling up of the corresponding intercostal spaces; displacement of the heart towards the opposite side; ægophony at the upper level of the fluid and skodaic resonance over the upper lobe in front when there is a moderate amount of fluid compressing the lower lobe behind. Of all these, that which most assists one in deciding whether or not there is much fluid present is the position of the cardiac impulse: if the latter is much displaced away from the side upon which the signs of fluid are, then the amount of fluid present must be considerable.

### Cardiac Impulse and its Errors.

Even this valuable sign, however, is open to several errors, of which the three most important probably are: First, that former pleurisy may have so bound the pleuræ or the pericardium to the chest wall or to the diaphragm that displacements within the thorax are no longer possible, or at least are reduced to a minimum. Secondly, the heart may itself become dilated so that the impulse is displaced when there may be no fluid present at all. Thirdly, the condition of the lungs as regards emphysema and the thickness of the chest wall as regards fat or muscle may make it difficult to determine the position of the cardiac impulse with any accuracy at all. It takes days or weeks, or even months for all the alveoli of a compressed lower lobe to re-expand and contain air again, so that the absence of resonance due to compression by a pleuritic effusion may persist for a long time after that effusion has gone. Probably the first things to return in a lung which, having been compressed, is now no longer so, though it has not yet re-expanded as to its alveoli, are the voice sounds; whereas these may have been absent all over the back of the lung at the time of the effusion; when the latter has been removed by tapping, or is clearing up, the voice sounds can be heard during the lung at a lower and lower level day by day even when dulness persists and when no vesicular murmur can be detected. The next physical sign to reappear in such cases probably is the tactile vocal fremitus; then vesicular murmur which, though still very much fainter than that upon the sound side, begins to be heard more and more distinctly over the previously compressed lung day by day until it can be heard down to the very base sometimes long before anything like good resonance has been restored. Voice sounds return first therefore, tactile vocal fremitus next, vesicular murmur third, and resonance last, during the process of the clearing up of a pleuritic effusion.

### The Evidence of Weight.

It is small wonder therefore that in many instances there is doubt as to whether the effusion is clearing up, remaining stationary, or increasing in amount, and as one further means of helping to decide this point it is rather surprising that the patient is not more frequently weighed if his general condition allows of it. It is most important of course that the circumstances of the weighing should be precisely comparable from day to day; it would be useless to compare a weight taken after breakfast and before micturition with another weight taken before breakfast and shortly after the emptying of the bladder. It is essential that the circumstances of the weighing should be similar day by day, and to this end it is probably best that the patient should be scaled before his breakfast and immediately after the emptying of his bladder in the morning; the clothes he wears either being removed subsequently and deducted from his total weight so as to obtain the patient's net weight naked, or else the same clothes such, for instance, as a nightgown, a dressing-gown, and a pair of slippers should be used from day to day; it will generally be possible to determine clinically whether the patient is losing weight from his disease, if, for instance, he has active phthisis or a malignant neoplasm.

As he improves in health, however, and as any fever subsides his body weight will naturally increase as he is allowed more food; in such instances, however, there will generally be sufficiently obvious cause for the increase or the diminution in weight respectively. Notwithstanding these sources of error there will manifestly be many patients in whom were it not for variations in the amount of fluid present, the weight ought to remain approximately constant and in whom therefore progressive diminution in the weight would be evidence of progressive decrease in the amount of fluid present, whilst conversely a progressive increase in the weight day by day would indicate progressive effusion which may require tapping to remove it. The method can generally be applied with ease in hospital wards; in private practice it may be less easy, but in suitable instances the difficulties can be surmounted, and the information obtainable is sometimes of considerable value, particularly if it obviates the necessity for the painful process of exploratory needling of the chest.

